# Cross-frequency phase-amplitude coupling in repetitive movements in patients with Parkinson’s disease

**DOI:** 10.1152/jn.00541.2021

**Published:** 2022-05-11

**Authors:** Ruxue Gong, Christoph Mühlberg, Mirko Wegscheider, Christopher Fricke, Jost-Julian Rumpf, Thomas R. Knösche, Joseph Classen

**Affiliations:** ^1^Department of Neurology, Leipzig University Medical Center, Leipzig, Germany; ^2^Method and Development Group Brain Networks, Max Planck Institute for Human Cognitive and Brain Sciences, Leipzig, Germany

**Keywords:** bradykinesia, noninvasive EEG, movement, phase-amplitude coupling, Parkinson’s disease

## Abstract

Bradykinesia is a cardinal motor symptom in Parkinson’s disease (PD), the pathophysiology of which is not fully understood. We analyzed the role of cross-frequency coupling of oscillatory cortical activity in motor impairment in patients with PD and healthy controls. High-density EEG signals were recorded during various motor activities and at rest. Patients performed a repetitive finger-pressing task normally, but were slower than controls during tapping. Phase-amplitude coupling (PAC) between β (13–30 Hz) and broadband γ (50–150 Hz) was computed from individual EEG source signals in the premotor, primary motor, and primary somatosensory cortices, and the primary somatosensory complex. In all four regions, averaging the entire movement period resulted in higher PAC in patients than in controls for the resting condition and the pressing task (similar performance between groups). However, this was not the case for the tapping tasks where patients performed slower. This suggests the strength of state-related β-γ PAC does not determine Parkinsonian bradykinesia. Examination of the dynamics of oscillatory EEG signals during motor transitions revealed a distinctive motif of PAC rise and decay around press onset. This pattern was also present at press offset and slow tapping onset, linking such idiosyncratic PAC changes to transitions between different movement states. The transition-related PAC modulation in patients was similar to controls in the pressing task but flattened during slow tapping, which related to normal and abnormal performance, respectively. These findings suggest that the dysfunctional evolution of neuronal population dynamics during movement execution is an important component of the pathophysiology of Parkinsonian bradykinesia.

**NEW & NOTEWORTHY** Our findings using noninvasive EEG recordings provide evidence that PAC dynamics might play a role in the physiological cortical control of movement execution and may encode transitions between movement states. Results in patients with Parkinson’s disease suggest that bradykinesia is related to a deficit of the dynamic regulation of PAC during movement execution rather than its absolute strength. Our findings may contribute to the development of a new concept of the pathophysiology of bradykinesia.

## Graphical abstract

**Figure:**
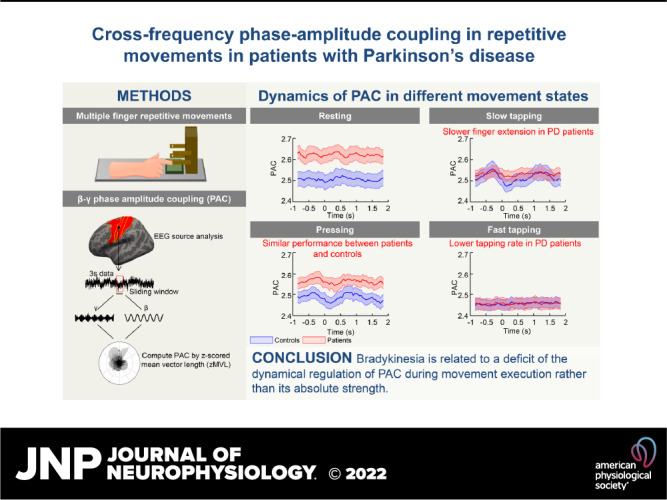

## INTRODUCTION

Brain rhythms at distinct frequencies interact with each other, enabling neuronal ensembles to flexibly bind together across different temporal and spatial scales. Although coupling among neural oscillations in different frequency bands may have functional significance for normal behavior (1–4), abnormal coupling has been associated with a variety of neurological disorders (5–7). It is widely held that exaggerated cross-frequency coupling between the phase of β oscillations and the amplitude of γ oscillations (β-γ PAC), as detected in cortical (8–10) or subcortical (11) resting state recordings of patients with Parkinson’s disease (PD), is closely involved in the pathophysiology of Parkinsonian motor impairment. Importantly, interventions that alleviate Parkinsonian motor impairment, such as deep brain stimulation (DBS) of the subthalamic nucleus (STN) or globus pallidus internus (GPi), and dopamine replacement therapy, also decrease enhanced resting state β-γ PAC in STN (12) and motor cortex (13–15). Increased resting β-γ PAC and β power have been found in subcortical nuclei of patients with PD (8, 11). However, at the cortical level, enhanced β-γ PAC at rest was more pronounced than enhanced β power (9, 13, 15). In addition, unlike the coupling between β phase and the relatively narrow γ band (around 250 Hz) found in subcortical nuclei of patients with PD (11, 12), the γ activity involved in the exaggerated cortical PAC were more broadband (50–200 Hz derived from ECoG and 50–150 Hz derived from scalp EEG) (8, 9, 14). Although this clearly suggests distinct underlying generative mechanisms, the wide range of γ oscillations in EEG and ECoG recordings could also result from the overlap of a large number of variably coupled local oscillators with different spectral and temporal properties (for an overview on the generative mechanism of γ, see Ref. 16). However, such a relationship between cortical PAC and motor impairment of PD may be difficult to interpret when determined in the absence of movement or impairment of movement.

It has been reported that cortical and subcortical β-γ PAC occurring at rest is suppressed during movement (8, 12, 15, 17). However, findings from previous studies are inconsistent regarding whether enhanced β-γ PAC in patients with PD remains enhanced or returns to normal during movements. On the one hand, PAC was found to be enhanced during movements in patients with PD. For example, movement-related PAC in patients with PD, derived from LFP recordings in the STN, was higher in off state than in medication on state (12). Also, at the cortical level (ECoG recordings in the sensorimotor areas), persistent enhancement of movement-related PAC in patients with PD was found, as compared with patients with a nonmovement-related neurological disorder (8). Furthermore, DBS-induced acceleration of movements and alleviation of bradykinesia during voluntary movement tasks have been associated with reduced cortical movement-related PAC (13, 15). On the other hand, PAC strength derived from scalp EEG did not differ between patients in the off-medication state and healthy controls during a verbally cued intermittent hand-opening/closing task (14). This inconsistency may reflect differences in the characteristics of concomitantly recorded movements. In addition, it is not clear from these reports whether movement-related PAC was derived from signals recorded during the execution of kinematically normal or abnormal movements (8, 14, 15). Because abnormalities of movement-related PAC may not become apparent until kinematic abnormalities occur, the lack of information on kinematics renders the involvement of PAC unknown, concerning the pathophysiology of movement disorders.

In addition, the temporal modulation of brain activity involved in motor control is of great significance. Changes between different motor states, such as perception, planning, and execution, are reflected in changes in neural signals from motor-related brain regions at specific transition times (18, 19). Recent evidence suggests dynamic motor control includes special “preparatory” states at motor state transitions (20, 21) that are essentially invariant to any specific muscle activity (18). However, previous attempts to relate β-γ PAC to motor impairment have not considered the transitions between movement segments. Thus, it is crucial to investigate neuronal dynamics, via the modulation of β-γ PAC, during the movement dynamics and in direct relationship to the motor behavior of patients with PD.

Bradykinesia (slowness of motor execution) and movement amplitude decrement during repetitive movements are cardinal kinematic abnormalities in patients with PD (22). Other movement parameters, such as grip force (23), patients’ natural tapping rate (24), or the ability to tap in sync with a pacemaker (25), do not reliably distinguish between patients and controls. The co-occurrence of pathological and intact motor behavior in patients with PD makes it possible to compare different movement types and elucidate the role of abnormal neuronal population dynamics underlying the movement impairment. If a physiological characteristic such as β-γ PAC is found to be abnormal during a kinematically normal motor behavior, it may be that the characteristic is insufficient for making the motor behavior abnormal. Conversely, if we find that a particular physiological abnormality is not present during a kinematically abnormal motor behavior then this abnormality may not be necessary for making the motor behavior abnormal.

Previous studies have demonstrated that the enhanced PAC at rest derived from ECoG recordings of patients with PD is localized in motor regions, including the premotor cortex (PMC) and the primary motor cortex (M1). In our previous paper (10), we have reported that enhanced PAC can also be detected in somatosensory areas, namely, the primary somatosensory cortex (BA3) and the primary somatosensory complex (BA1&2), via EEG source localization techniques. These four brain regions have also been reported to be involved in motor control (26, 27). Here, we report our analyses of oscillatory EEG activity in these regions during different types of voluntary repetitive movement tasks in patients with PD. Certain tasks were chosen specifically to elicit the cardinal motor signs employed in clinical examination of PD. Our results challenge the view that bradykinesia is merely a consequence of the strength of cross-frequency coupling. Rather, they suggest that bradykinesia might be better understood as a deficit of the dynamic regulation of that coupling.

## MATERIALS AND METHODS

### Experimental Design

#### Participants.

Nineteen patients with PD (6 females, mean age: 60.9 ± 10.8 yr) and twenty age- and sex-matched healthy controls (8 females, mean age: 62.6 ± 7.9 yr) were recruited and included in the analysis of this study. Patients’ characteristics have been described in detail previously (10) and are also provided in Supplemental Table S1 (https://doi.org/10.5281/zenodo.6535236). There was no significant age difference between patients and controls (*P* = 0.527). The experiment was carried out during a practically defined “off-medication” state (at least 12 h overnight withdrawal of Parkinsonian medication). Before starting the experiment, clinical scores indicating the motor impairment of each patient were estimated by a trained neurologist, using the International Parkinson and Movement Disorders Society Unified Parkinson’s Disease rating scale part III (MDS-UPDRS III). All participants were right-handed, as confirmed by the Edinburgh Handedness Inventory (28). Written informed consent was obtained from all patients and controls according to the protocol approved by the local Ethics Committee at the Medical Faculty of Leipzig University (Reference No. 147/18-ek).

#### Movement recording.

**Figure 1.**
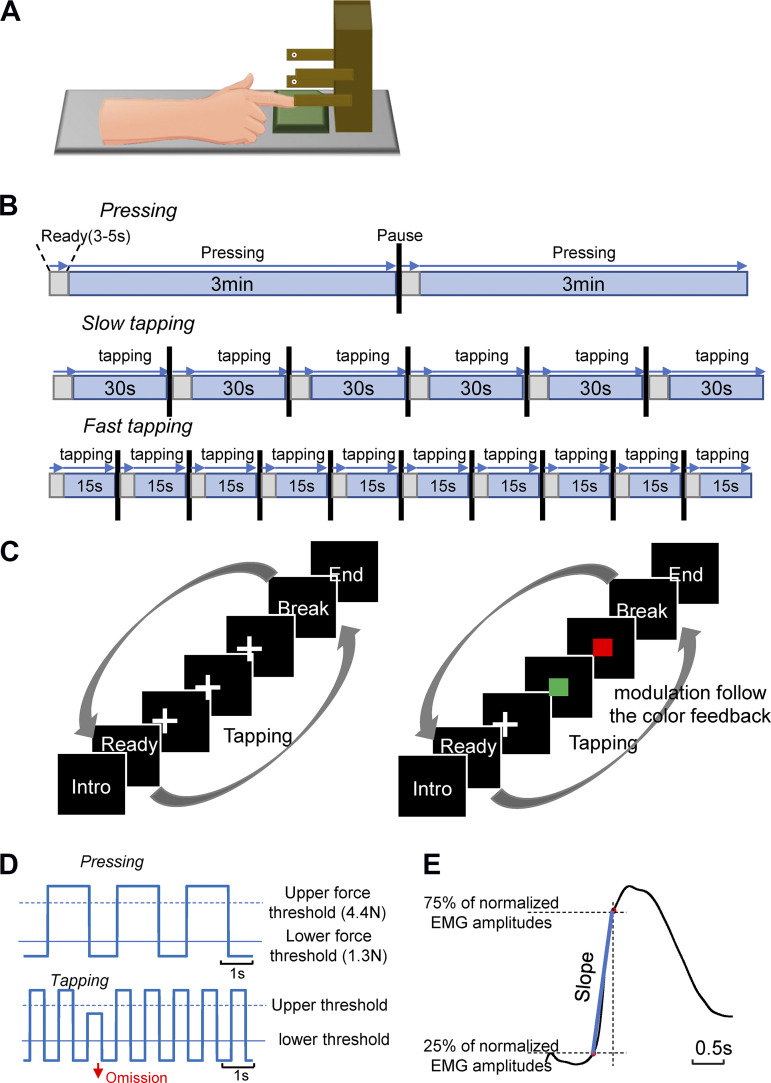
Experimental design. *A*: schematic diagram of the experimental setup. The device for recording repetitive movements consisted of a transducer (green plate), recording the press force and two photoelectric sensors (white) reporting the height of the extended index finger. *B*: experimental protocol for the three movement tasks. Gray areas represent the short periods when subjects prepared to perform the task. Blue areas represent the active movement periods. *C*: experimental design of the tapping tasks. There were two conditions for each tapping task. *Left*: subjects performed repetitive tapping while looking at the fixation cross. *Right*: after each tapping cycle, subjects received color feedback indicating whether the extended index finger had reached the upper level. A green square indicated that the level of the preceding tapping movement had been at or above the upper photoelectric sensor. A red square indicated that the tapping height had been below the upper photoelectric sensor. Subjects were instructed to maximize green feedback by performing sufficiently extended tapping movements. *D*: schematic representation of the force and amplitude trajectories during pressing (*top curve*) and slow tapping (*bottom curve*). In pressing, the peripheral movement onset was defined as the moment when the force signal exceeded the lower force threshold (1.3 N). No force signals were resolved above the upper threshold of 4.4 N. During tapping, the peripheral movement onset was defined as the moment when the index finger was extended above the light beam of the lower photoelectric sensor. An omission was recorded if the index finger was not extended high enough to reach or cross the light beam of the upper sensor. *E*: estimation of the EMG slope. This was defined as the slope of the line (blue) connecting the 25% and 75% percentile (red dots) of the normalized EMG signal closest to the movement onset of tapping.

Pressing and tapping movements (see *Movement tasks*) were recorded by a custom-made device consisting of a force transducer and two photoelectric beam sensors (Fig. 1*A*). The digital signal from the force transducer was used to define the peripheral time of movement onset and offset of the pressing task. For that task, the peripheral movement onset and offset of pressing were defined as the moments when the force exceeded or fell below a threshold of 1.3 N, respectively. A threshold of 1.3 N was empirically derived from pilot studies. This threshold was chosen such that it reliably fell into the interval between the force values at rest and constant pressure for all subjects. The maximum force that the transducer could detect was 4.4 N.

The lower photoelectric sensor was placed at a height just above the finger when placed on the pressure sensing board. The upper photoelectric sensor was placed at the height of the extended index finger in a position parallel to the table. During the tapping tasks, the peripheral movement onset of index finger extension was defined as the time when the light beam of the lower photoelectric sensor was interrupted by the extended index finger. The upper photoelectric sensor was used to signal the elevation level of the extended index finger during tapping. During tapping movements, omissions were registered when the index finger did not reach the upper photoelectric sensor level. Figure 1*D* illustrates how information concerning movement events was determined during the pressing and tapping tasks. The peripheral movement onsets recorded online were visually verified afterward for mechanical or performance errors.

#### Movement tasks.

Participants were asked to perform three different voluntary movement tasks involving repetitive index finger actions: pressing, tapping at slow speed, and tapping at fast speed. These tasks were chosen to represent behaviors that have different probabilities of triggering motor impairment in patients with PD, as reported in previous literature (17, 23, 29). We tested the performance of the patients on the side most severely affected by the disease, as indicated by the bradykinesia hemibody scores (10 patients on the left and 9 patients on the right) in MDS-UPDRS III. In healthy controls, the side for performing the movement tasks was pseudorandomly chosen to eventually match the respective subsample sizes of patients (10 controls on the left and 10 controls on the right). Before starting each trial, participants were asked to place their arm on an armrest and place their index finger on the pressure sensing board. They were asked to start the index finger movements as soon as a white cross appeared at the center of the computer screen.

The experimental protocol is illustrated in Fig. 1*B*. In the pressing task, subjects were asked to perform self-initiated press and release movements using the index finger at a comfortable rate (2 trials of 3 min each). In the slow tapping tasks (6 trials of 30 s each, two blocks), subjects were instructed to tap at a slow rate (instruction “tap at your own comfortable speed”). In the fast tapping tasks [10 trials of 12 s or 15 s each, two blocks (9 patients and 11 controls tapped for 12 s/trial in the second block of fast tapping tasks)], subjects were instructed to tap at their fastest speed (instruction: “tap as fast as possible”). During the slow and fast tapping tasks, subjects were asked to tap with their index finger maximally extended. Between trials and tasks, subjects were allowed sufficient time to rest, to minimize fatigue. Two different conditions (blocks) were designed for each of the tapping tasks (Fig. 1*C*). These conditions differed in the absence (FB-) or presence (FB+) of feedback as to whether the index finger extension had met the upper-level criterion, as detected by the upper photoelectric light beam (Fig. 1*C*). In FB+, subjects were instructed to increase their index finger’s height according to the feedback, if necessary. The feedback consisted of a colored square displayed on the screen after each tap. The color indicated whether the index finger extension had reached the upper threshold (Fig. 1*C*, *right*). Hence, the motor tasks consisted of five conditions in the following order: repeated press at a slow rate, slow tapping without feedback, slow tapping with feedback, fast tapping without feedback, and fast tapping with feedback.

#### Motor performance metrics.

In each tapping task, motor performance was indexed by tapping rate, tapping variability, and the completion ratio. The mean tapping rate was calculated in each tapping task as the number of all index finger extensions crossing the lower light beam per second. The tapping variability was calculated as the standard deviation of the normalized movement intervals across a trial. The movement intervals were obtained as the time intervals between adjacent movement onsets. Concerning the variation of movement rates between subjects, time intervals were normalized to the maximum interval in a trial. We then computed the mean tapping variability across trials, per task, for each subject. The completion ratio was computed as the ratio of index finger extensions reaching the level of the upper photoelectric sensor divided by the total number of index finger extensions.

To estimate the decrement, the trials were first distributed into 12 time bins in the slow and the fast tapping tasks (the first 12 s). Decrement was then defined as the decrease of the completion ratios from the first to the twelfth time bin in a trial. We evaluated the decrement by estimating the effect of time bin on the completion ratios in a mixed model [completion ratios ∼ 1 + time bin + random(1 | subjectID)]. To compare the decrements in the slow and fast tapping tasks over identical time spans, we also calculated completion ratios for each 1-s time bin during the first 12 s of slow tapping.

### Signal Recording and Processing

#### EEG signal recordings.

We recorded high-resolution (24 bit) EEG signals with 64-channels (eego mylab, ANT Neuro, the Netherlands) at a sampling rate of 2 kHz. Vertical electrooculography was also recorded for removal of eye movement components. Bipolar EMG of the first dorsal interosseous (FDI) muscle was recorded from the hand that performed the repetitive movement tasks. Individual positions of the EEG electrodes and fiducial markers were acquired by a 3-D optical digitization system (EEG Pinpoint, Localite, Germany) before EEG recordings. Data were recorded during the 5-min resting period and the subsequent repetitive movement tasks (as mentioned in *Movement tasks*).

#### EEG signal preprocessing.

All EEG signal preprocessing procedures were done in the EEGLab Toolbox under a common pipeline. Channels that contained large long-term artifacts were excluded from the subsequent analysis after raw data of all the channels were demeaned. We applied high-pass filtering at 0.5 Hz to remove any slow drift. Independent component analysis (ICA) was applied and manually assessed to remove components that contained eye movement artifacts, channel noise, line noise, ECG artifacts, and major muscle artifacts. Artifacts from transitory muscle activities that contaminated the EEG signals were visually detected and marked in the raw data. For EEG source analysis, the data was re-referenced from the original reference (CPz) to the average of all channels. To eliminate the high-frequency interference, we also applied a 300-Hz low-pass filter [EEGLab default finite impulse response (FIR) filter]. Subsequently, all datasets were segmented into epochs of 3-s duration (−1 s to 2 s relative to the peripheral movement onset). To ensure equal treatment of the resting dataset, we randomly generated and inserted 150 fake markers into the original raw datasets, and the epoching information was saved based on these fake markers. For the fast tapping task, we only used every second movement onset marker to reduce the overlap between epochs. The number of recorded movement cycles varied between the different movement tasks, with the least number of repetitions performed in the pressing task. To obtain a comparable number of movement cycles across conditions, we randomly selected trials in resting, slow tapping, and fast tapping conditions, such that their numbers matched that derived from the pressing task. The average number of epochs was 106, and no significant differences (*P* > 0.05, before correction) were found between patients and controls or across conditions (as detailed in Supplemental Table S2).

#### EMG signal processing.

The EMG signals were first preprocessed together with the EEG signals. They were demeaned, high-pass filtered at 0.5 Hz, cleared from visually detected EEG/EMG artifacts, and broadband pass filtered from 5 Hz to 200 Hz. Notch filters at 50 Hz and its harmonics were applied to reduce interference from environmental noise. After rectification, EMG signals were segmented into 3-s epochs (−1 s to 2 s) and aligned with the movement onset as defined in *EEG signal preprocessing*. EMG signals were smoothed by applying a 5th order Butterworth low pass filter at 5 Hz to the rectified signals (30). For each subject, the EMG signals were averaged across epochs. Based on the mean EMG signal of each subject, we calculated the EMG slope as the slope between the 25% and 75% percentiles of the normalized EMG amplitudes close to the movement onset (as shown in Fig. 1*E*). EMG signals were *z*-score normalized before computing the EMG slope to diminish individual variability of the EMG amplitudes.

#### Region-based source analysis.

We applied the EEG source analysis procedure introduced in our previous paper (10). The raw data from EEG sensor signals were projected onto the cortical surface employing individual head models and a linearly constrained minimum variance beamformer method (31) to obtain the source signal in each specific brain region. For each source region, the signal (number of voxels times number of time steps) was ICA decomposed as proposed by Jonmohamadi et al. (32). That is, the resulting components, representing regional source patterns with mutually independent time courses, were ordered according to their explained variances and a minimal set that explained 95% of the data variance was selected. This procedure helped eliminate noise and limit the number of independent sources patterns in each region. This study investigated the four brain regions that we previously found to show statistically enhanced PAC in patients with PD compared with controls (10). These regions were PMC, M1, BA3, and BA1&2, which were defined with reference to the multimodal parcellation by Glasser et al. (33). Details are described in the Supplemental Method “*Region-based source analysis*.”. Therefore, in each of the six conditions, for each subject, we obtained between 8 and 13 ICA components for each of the four brain regions. Detailed information regarding the average number (means ± SD) of components of each region across patients/controls in each of the six conditions is provided in Supplemental Table S3.

#### Calculation of movement-related PAC.

To investigate movement-related PAC, both by comparing it under different conditions and by tracking its dynamics over a movement cycle, we applied time-resolved PAC analysis. The source signals (i.e., component time courses) were first filtered into β (13–30 Hz) and γ (50–150 Hz) bands. The width of γ frequency was defined based on previous literature (9, 14). Subsequently, the Hilbert transform was applied to extract the phase of the β band and the amplitude of the γ band. Then, for each 3 s epoch in a component, we computed PAC in successive windows (300 ms, corresponding to 4–10 cycles for the β activities) shifted by 50 ms time steps. We applied the normalized mean vector length (MVL) method (34, 35), in which the normalization factor is the square root mean of the γ amplitudes in a time window. As the time window was rather short, which might lead to inaccurate PAC estimation, the *z*-score of the MVL (zMVL) was calculated using the mean and standard deviation of 200 surrogates, created by recombining the instantaneous phase and randomized shuffled amplitudes. zMVL values not larger than 1.96 (equivalent to 95% confidence interval) were assigned a value 0. Therefore, for each 3-s epoch in a component, we had 55 zMVL values with 50 ms time resolution.

For each subject, we calculated pairwise zMVL values among ICA component pairs within each region as introduced previously (10). That is, the β phases and γ amplitudes were not only extracted from the same component, but also from different components. The method for computing n ICA components in a region was introduced in “*Region-based source analysis*.” In brief, we calculated the epochs of the time series of zMVL values among *n* × *n* component pairs in a region. We then computed a single time series by computing the weighted average of zMVL values across trials and *n* × *n* component. The weights for averaging were defined as the percentage of variance accounted for by each component pair in the region.

##### State-related PAC.

To compare PAC across conditions and groups, we computed the mean PAC value by averaging across the 55 time points of 3-s epochs for each subject in each condition.

##### Fluctuations of movement-related PAC.

To estimate the fluctuation of PAC over time in different tasks, we calculated the coefficient of variance (CoV) of the movement-related PAC for each task and for each subject. CoV was calculated as the ratio of the standard deviation to the mean across 55 time points of averaged PAC for each subject.

##### Dynamic PAC estimation.

Movement cycles varied between subjects, and movement states within a movement cycle had different durations. Therefore, we first determined the time points when movement transitions occurred between different movement phases in each subject. Data were then aligned with these transition points to enable the averaging of dynamic PAC (dynPAC) values associated with movement transitions and to assess PAC changes across transitions between movement states. We subdivided PAC values of each movement cycle into five periods separated by four movement transition points defined at the level of the cortex (cortical transition points). The cortical transition points were defined to estimate the timing of the transitions at the cortical level with optimal accuracy. They were derived from the peripheral movement transition points which were measured by kinematic signals. In brief, we computed the cortical transition points by translocating the timing of the peripheral movement transition points, taking into account the mechanical delays, electromechanical delays and corticomuscular conduction time. The details for the determination of the transition points are described in the Supplemental Method section “*Definition of 4 transition points of a movement cycle.*”.

Therefore, for the pressing task, the PAC values in the movement epochs were grouped into five periods at cortical level. These included: P1 (prepress onset period: from 200 ms before the movement onset to the movement onset), P2 (postpress onset period: from the movement onset until the end of the force build-up), P3 (sustained press period: from the end of the force build-up to the start of the force release), P4 (release period: from the start of finger release to the movement offset), and P5 (postoffset period: from the movement offset to 200 ms after the movement offset).

In addition, for the slow tapping task, the five periods at the cortical level were defined as: T1 (pre-extension onset period: from 200 ms before the movement onset to movement onset), T2 (postextension onset period: from the movement onset to the end of finger extension), T3 (extension period: during which the index finger remained extended at or above the upper photoelectric sensor), T4 (index finger flexion period: from the start of finger flexion to the movement offset), and T5 (post-flexion offset period: from the movement offset to 200 ms after the movement offset).

The zMVL values in the single movement cycle were then grouped in the 5 periods of pressing and slow tapping tasks, respectively. We then averaged the zMVL values in each of the periods across trials for each subject.

#### Power spectral density.

The power spectral density (PSD) was calculated by the Welch method implemented in MATLAB. To be comparable with the movement-related PAC, the PSD was calculated in 300 ms shifted windows with 50 ms time steps in 3-s epochs (Hann window, frequency resolution 1 Hz) for all conditions. Then, PSD was transformed to base 10 logarithmic power for group comparison and presentation. The power was normalized by subtracting the mean power across all time points and trials from 4 to 300 Hz (excluding 50 Hz and its harmonics) to account for the intersubject variability.

### Statistical Analysis

All analyses were performed in the brain regions contralateral to the hand that the subject used to perform the movement tasks. We mainly applied ANOVA tests to examine the main and interaction effects of the analysis factors. Since our data were not always Gaussian distributed and of equal variance, we applied a nonparametric ANOVA provided by the ARTool package (Aligned Rank Transform for nonparametric factorial ANOVAs) on R (36). This package applies the aligned rank transform to the responses of each main or interaction effect in the designed model and then runs a factorial ANOVA (type III Wald F tests with Kenward–Roger degrees of freedom) on the transformed data. For post hoc tests, we applied a nonparametric Wilcoxon rank-sum test for between-group comparisons and Wilcoxon signed-rank test for within-group comparisons across conditions and movement transitions. We performed post hoc tests after significant ANOVA effects were found. Detailed information on the selection of post-hoc tests is specified in the relevant Result sections. Moreover, we computed Spearman correlations between PAC, PSD, performance parameters, and clinical severity scores. The false discovery rate (FDR) correction was applied in multiple tests to avoid type I errors in null hypothesis testing.

## RESULTS

### Behavioral Analysis

**Figure 2.**
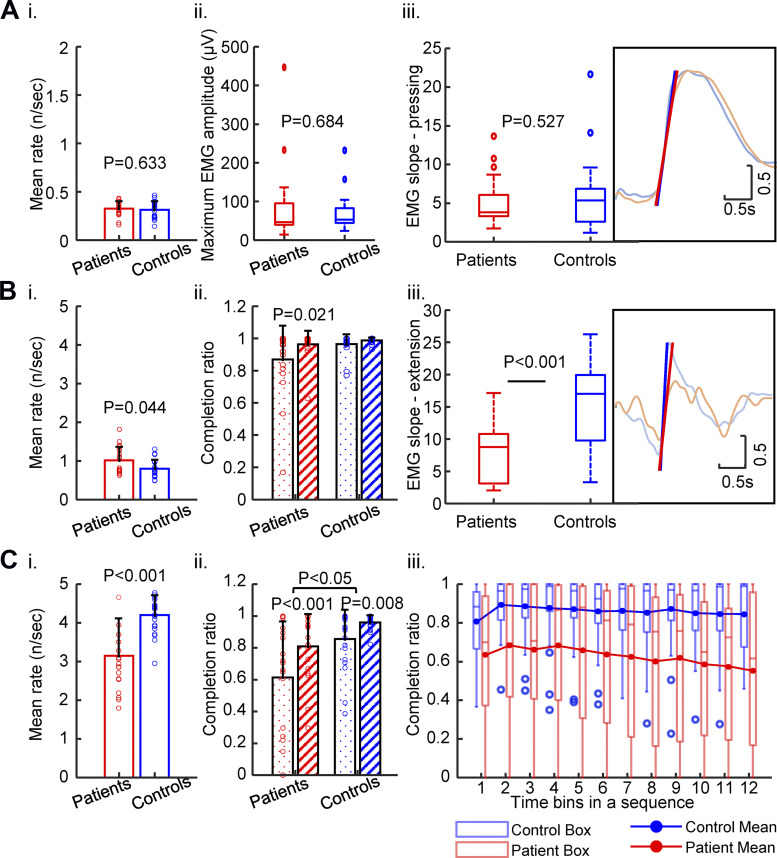
Selected performance parameters of the repetitive movement tasks. *A*: pressing: *i*) rate of repetitive pressing actions, *ii*) maximum amplitude of EMG recorded from FDI muscle during pressing, *iii*) slope of EMG activity in FDI muscle during the build-up of press force. *Inset*: averaged EMG curves of patients (red) and controls (blue). Group differences were estimated by Wilcoxon rank-sum tests. All *P* values were not significant even before correction. *B*: slow tapping: *i*) rate of repetitive slow tapping actions, *ii*) completion ratio of index finger extensions meeting the upper amplitude criterion (dotted: without feedback, hatched: with feedback), *iii*) slope of EMG activity in FDI muscle upon index finger extension. *Inset*: averaged EMG signals of patients (red) and controls (blue). Note the reduced EMG slope in patients. *P* values were adjusted by FDR correction in the respective post hoc tests. Only the adjusted *P* values below 0.05 are shown in the figure. *C*: fast tapping: *i*) rate of repetitive fast tapping actions, *ii*) rate of index finger extensions meeting the upper amplitude criterion (no visual feedback about the level of index finger extension), *iii*) box plot combined with mean lines showed decline of fraction of extension movements meeting the upper amplitude criterion across sequential 1-s time bins in fast tapping task without feedback. *P* values were adjusted by FDR correction in the respective post hoc tests. Only the adjusted *P* values below 0.05 are shown in the figure. The tests included 19 patients (6 females, mean age: 60.9 ± 10.8 yr) with and 20 healthy controls (8 females, mean age: 62.6 ± 7.9 yr). FDI, first dorsal interosseous; FDR, false discovery rate.

The behavioral analysis provided evidence for slowed motor performance in the patients, which varied by task and observed parameters (Fig. 2). In the pressing task, patients and controls performed press-release actions at a similar rate (patients, 0.33 ± 0.08/s; controls, 0.31 ± 0.09/s; Fig. 2*Ai*). The maximum EMG amplitude did not differ between patients and controls (Fig. 2*Aii*). Likewise, the EMG slopes (see Fig. 1*E*) regarding either force build-up or release were similar between patients and controls (force build-up in Fig. 2*Aiii*; releasing, *P* > 0.99, data not shown).

In the tapping tasks, we applied a two-way mixed ANOVA with the factors group and feedback on the three performance parameters (tapping rate, tapping variability, and completion ratio), as shown in Table 1. In slow tapping, the tapping rate was slightly higher in patients (mean rate: 1.01 ± 0.35/s) than in controls (mean rate: 0.80 ± 0.23/s, Fig. 2*Bi*). Rate increased in both groups with visual feedback, probably through an effect of pacing by the visual feedback (Table 1). The tapping variability was affected neither by group nor by feedback (Table 1). Regarding the mean completion ratio, which indicates the mean percentage of taps reaching the amplitude criterion in a task, we found a significant interaction effect between group and feedback (Table 1). Post hoc testing (Fig. 2*Bii*) revealed no significant differences between groups, although the completion ratio increased with visual feedback only in patients. More interestingly, the EMG slope at tapping onset (index finger extension) was significantly lower in patients compared with controls (Fig. 2*Biii*).

**Table 1.** Nonparametric two-way mixed ANOVA in performance metrics of tapping tasks

	Group (2 Levels)	Feedback (2 Levels)	Group × Feedback
Slow tapping task			
Tapping rate	***F* (1,37) = 44.68, *P* = 0.044**	***F *(1,37) = 14.63, *P* = 0.001**	*F *(1,37) = 0.47, *P* = 0.596
Tapping variability	*F *(1,37) = 0.18, *P* = 0.667	*F *(1,37) = 1.46, *P* = 0.281	*F *(1,37) = 0.11, *P* = 0.745
Completion ratio	***F *(1,37) = 6.74, *P* = 0.020**	***F *(1,37) = 14.17, *P* = 0.001**	***F *(1,37) = 8.91, *P* = 0.017**
Fast tapping task			
Tapping rate	***F *(1,37) = 18.22, *P* < 0.001**	***F *(1,37) = 9.46, *P* = 0.006**	*F *(1,37) = 0.91, *P* = 0.520
Tapping variability	***F *(1,37) = 14.33, *P* = 0.002**	*F *(1,37) = 0.68, *P* = 0.416	*F *(1,37) = 3.12, *P* = 0.172
Completion ratio	***F *(1,37) = 10.43, *P* = 0.005**	***F *(1,37) = 67.73, *P* < 0.001**	***F *(1,37) = 8.65, *P* = 0.017**

The factor group contains two levels: patients and controls; the factor feedback contains two levels: without and with feedback.

*P* values of each effect were adjusted according to FDR correction across the six ANOVA tests. All significant values (adjusted *P* < 0.05) after correction are marked in bold.

In the fast tapping tasks, patients showed a lower tapping rate (mean rate: 3.15 ± 0.97/s, Fig. 2*Ci*) and higher tapping variability (Table 1) than controls (mean rate: 4.20 ± 0.51/s). Feedback improved the tapping rate but not the tapping variability in both groups (Table 1). For the mean completion ratio, ANOVA revealed a significant interaction effect between group and feedback (Table 1). As shown in Fig. 2*Cii*, the completion ratio was lower in patients than in controls, both with and without feedback.

The decrement of tapping amplitude, as indexed by the decrease of completion ratio over a trial duration, was evaluated for tapping without visual feedback. We computed the mixed-effects model “completion ratio ∼ 1 + time bin + random(subjects),” separately for patients and controls. In slow tapping, the effect of time bin on the completion ratio was significant in patients, due to a decline over time [*t*(226) = −4.69, *P* < 0.001, FDR corrected] when 30-s epochs were used. The effect was no longer significant [*t*(226) = −1.58, *P* = 0.138, FDR corrected] when only considering the first 12 s of a trial, to facilitate comparison with the fast tapping task. Controls did not exhibit any decrement of amplitude in either case (data not shown). In fast tapping, evaluating the decrease of the completion ratio along the sequence of time points, we found a significant effect of time bin on the decrease of the completion ratio in the patients [*t*(226) = −5.45, *P* < 0.001, FDR corrected].

In summary, whereas in the pressing task all performance metrics of patients were similar to those of the control subjects, in slow tapping, the evidence for abnormal slowness was limited to the speed of muscle recruitment, whereas the tapping rate was unaffected. In fast tapping, the rate was reduced and a decrement was evident.

Given the nature of the movement tasks, the number of movement metrics derived from the tapping task was higher than from the pressing tasks. This may have increased the likelihood that we would find differences by chance in certain movement metrics for tapping compared with pressing. To address this issue, we additionally performed an ANOVA with the factor group (patients, controls) for the variable EMG slope. We found a significant interaction effect between Group and Task [*F*(1,37) = 17.97, *P* < 0.001], which suggests that the behavioral comparison between groups was different in the pressing and slow tapping tasks in terms of the EMG slope.

We then investigated the relationship between various abnormal performance metrics in the tapping tasks and the clinical severity of motor impairment in patients as indexed by the hemi-body bradykinesia and rigidity scores from the MDS-UPDRS III. We tested the correlations between clinical scores and four performance parameters: EMG slope, decrement for slow tapping task and the mean tapping rate, and decrement for fast tapping task. We expected these measures to be similar to the motor impairment as assessed in the qualitative clinical examination. Of all the parameters, the correlation between EMG slope at tapping onset in slow tapping and the hemibody bradykinesia and rigidity scores was marginally significant (ρ = −0.55, *P* = 0.056, FDR corrected). This supports the assumption of the clinical validity of this parameter. There were no correlations between the clinical score and the decrement computed as the difference of the completion ratios between the 1st and 12th time bins in either the slow or the fast tapping task.

### Similar State-Related PAC during Tapping Tasks between Patients with PD and Controls

**Figure 3.**
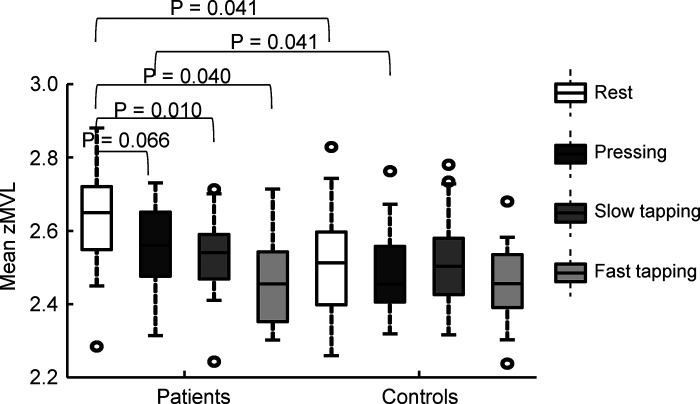
State-related PAC for the different tasks, averaged across four regions. We averaged zMVL values across the *n* × *n* pairwise PAC matrix and over the whole 3-s time series. A three-way nonparametric mixed ANOVA (PAC ∼ group × task × region) showed significant interaction effects between group and tasks. Post hoc tests of the group × task interaction effect showed significant differences between the resting state and all three movement tasks in the patients. Note that state-related PAC differed between patients and controls in resting state and during pressing. The tests included 19 patients (6 females, mean age: 60.9 ± 10.8 yr) with and 20 healthy controls (8 females, mean age: 62.6 ± 7.9 yr). *P* values were adjusted by FDR correction across 10 post hoc comparisons (4 between-group tests and 6 within-group tests). The adjusted *P* values below 0.1 are shown in the figure. FDR, false discovery rate; PAC, phase-amplitude coupling; zMVL, *z* score of the normalized mean vector length.

PAC values during the movement were calculated as zMVL as described in the materials and methods. We examined whether PAC averaged over the whole movement period (3-s time series) of each different condition (state-related PAC) was modulated by different tasks in patients and controls. First, zMVL values were averaged across the pairwise matrix of ICA components (*n* × *n* components), for each subject in each condition, as described in materials and methods, *Calculation of movement-related PAC*. To reduce the number of factors, we first established that the visual feedback did not modulate the strength of state-related PAC [3-way ANOVAs with factors Group (patient, controls), region (4 levels: PMC, M1, BA3, BA1&2) and feedback (2 levels: with or without visual feedback of tapping amplitude) for slow and fast tapping tasks]. The factor feedback showed neither an interaction nor main effects in either the slow [*F*(1,37) = 0.26, *P* = 0.616] or the fast tapping tasks [*F*(1,37) = 0.04, *P* = 0.840]. Therefore, in the following analysis, we pooled the two feedback conditions together. A three-way (group, task, and region) mixed ANOVA performed on the zMVL values averaged over the entire 3-s epochs suggested that state-related PAC was modulated differently in patients and controls by the tasks [group × task; *F*(3,111) = 4.69, *P* = 0.004; Fig. 3]. Next, we were interested in group differences across tasks and in the question of whether PAC was altered during movement versus rest. Post hoc Wilcoxon rank-sum testing for between-group comparisons revealed enhanced PAC in patients compared with controls in the resting state, in agreement with previous findings (10). PAC was also enhanced in patients in the pressing task, but not in any of the tapping tasks (all *P* values > 0.5). Post hoc testing for within-group comparisons showed that state-related PAC in patients was reduced during all active movement tasks compared with rest (Fig. 3). In controls, we found no significant reduction of state-related PAC during any movement tasks compared with the resting state. The ANOVA also revealed that PAC differences among the conditions were affected by the motor control area [task × region; *F*(9,333) = 2.64, *P* = 0.006]. However, since we found no interaction effects among the three factors [task × region × group; *F*(9,333) = 0.40, *P* = 0.933], the effect of brain regions was unlikely to have affected the differences between groups.

In addition, we also performed separate statistical tests on the PAC averaged from identical components and PAC from interactions between different components, respectively. The results were similar to that of the state-related PAC averaged across the whole PAC matrix (Supplemental Fig. S1).

In summary, the abovementioned findings show differences between the resting state and movement state-related PAC, especially in patients. These results were not in direct congruence with the behavioral results. In the pressing task, state-related PAC differed between patients and controls, whereas motor behavior was similar. In contrast, in the tapping tasks, state-related PAC was remarkably similar between patients and controls, whereas motor behavior was different. This suggests that abnormal enhancement of PAC per se is unlikely to be directly related to motor impairment.

### Dynamics of PAC during Transitions between Different Movement States

**Figure 4.**
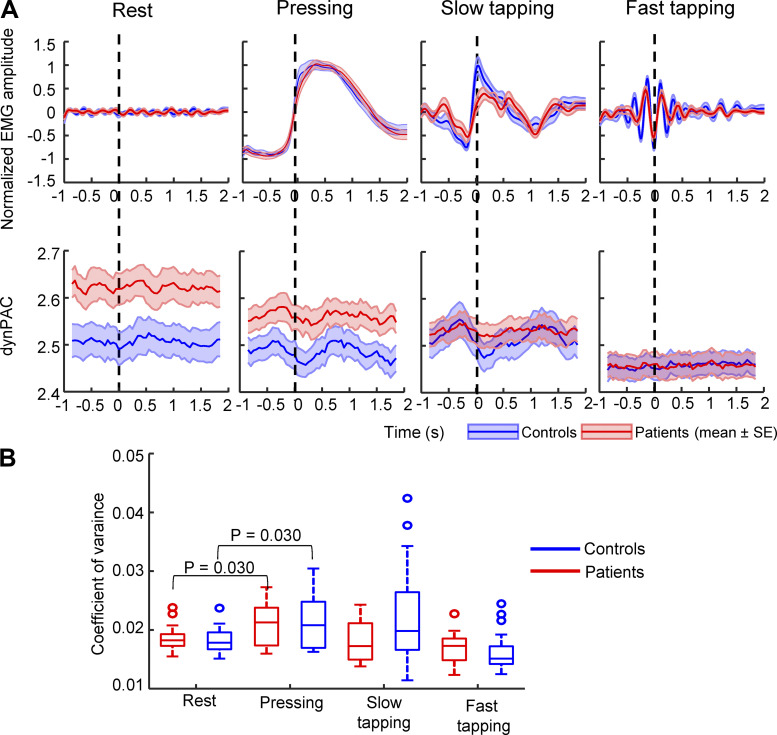
Fluctuation of PAC in the 3-s time series. *A*: PAC dynamics in the four conditions. *Top*: rectified *z*-score normalized EMG (means ± SE), recorded from first dorsal interosseus muscle, averaged after alignment to arbitrary time points (rest) or to peripheral movement onset (pressing, slow and fast tapping). *Bottom*: PAC (means ± SE) averaged after alignment to arbitrary time points (rest) or to peripheral movement onset (pressing, slow and fast tapping). *B*: box plot shows coefficient of variance of PAC across the four conditions. The tests included 19 patients (6 females, mean age: 60.9 ± 10.8 yr) with and 20 healthy controls (8 females, mean age: 62.6 ± 7.9 yr). *P* values were adjusted by FDR correction across 10 post hoc comparisons (4 between-group tests and 6 within-group tests). The adjusted *P* values below 0.1 are shown in the figure. dynPAC, dynamic PAC; FDI, first dorsal interosseous; FDR, false discovery rate; PAC, phase-amplitude coupling.

We then considered the possibility that the dynamic modulation of PAC might be more directly related to the underlying pathophysiology of motor impairment than its absolute level. We first visualized dynamic PAC and EMG activity recorded from the FDI muscle, aligned with the peripheral movement onset (Fig. 4*A*). Alignment to movement onset confirmed that, in patients, PAC was generally reduced in all movement tasks compared with the resting state, as shown in the previous paragraph. In addition, it became evident that in controls, the PAC values in the pressing and slow movement tasks were markedly modulated along the movement cycle. In contrast, this modulation was considerably less pronounced in the resting state and during fast tapping. For pressing and slow tapping, PAC rapidly and markedly declined around movement onset after a brief peak and then rebounded again. Modulation appeared to be less marked in patients.

To statistically assess the degree of fluctuation of movement-related PAC for each task quantitatively, we first computed the coefficient of variance (CoV) across the time series. A two-way mixed ANOVA test (CoV ∼ group × task) showed a significant interaction [*F*(3,111) = 3.07, *P* = 0.031], which indicated that PAC varies differently between groups. In pressing, post hoc testing revealed that the fluctuation was stronger for both patients and controls compared with the resting state (Fig. 4*B*). In slow tapping, the fluctuation was only larger than in the resting state in controls before FDR correction (Wilcoxon signed-rank, *P* = 0.033). Since the resolution of the PAC calculation does not permit assessment of modulation across very short movement cycles, fluctuation of PAC in fast tapping task (tapping rate around 4 Hz) across a movement cycle must be interpreted with caution. The larger time-series variance of PAC in pressing and slow tapping tasks compared with the resting state of controls may hint at the possibility that PAC may be modulated at particular movement events. However, direct comparisons of CoV between patients and controls did not reveal any significant differences in the tasks (Fig. 4*B*), even though we noticed different variability of PAC between patients and controls in Fig. 4*A* This may be because CoV measurement can only evaluate the general variability of PAC over time, and lost power to detect the changes at specific transition times. Therefore, we subsequently investigated the modulation of movement-related PAC across transitions between different movement states in the pressing and slow tapping tasks (“dynamic PAC”).

**Figure 5.**
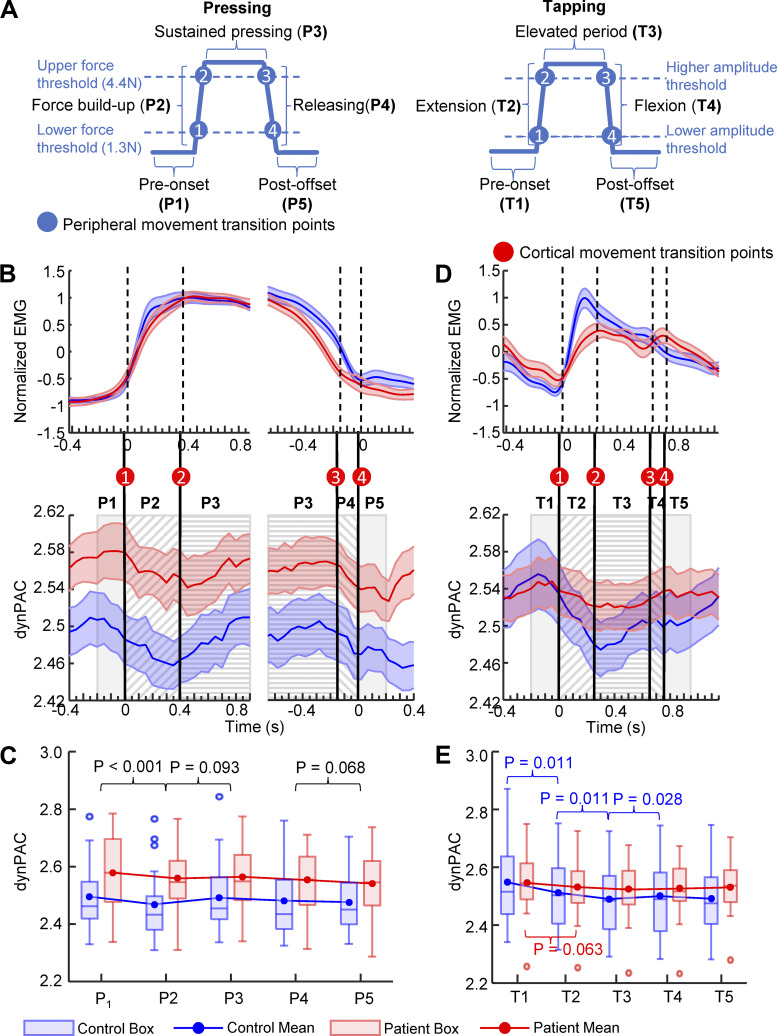
PAC dynamics across movement transitions. *A*: definition of five periods of movement based on four movement transition points in the pressing task (*left*) and the slow tapping task (*right*). We considered four kinetic (force threshold for pressing) or kinematic (amplitude criterion for tapping) events along the movement cycle as the peripheral movement transition points. The movement transition points (displayed in *B* and *D*) at the level of the cortex were determined by taking into account the mechanical delays, electromechanical delays and the corticomuscular conduction time to estimate the timing of the transition times. *B*: pressing task. Dynamic PAC resulting from averaging after alignment with the transition points on the (*left*) cortical movement onset (#1) and (*right*) cortical movement offset (#4). The timing of the other two cortical movement transition points (*left*: transition point #2, right: transition point #3) was determined based on the averaged durations of periods (*left*: P2 and P3, right: P3 and P4) across subjects. Note similar PAC modulation patterns for press onset and offset. *C*: box graph combined with mean lines of movement-related dynamic PAC in the 5 periods of a single pressing cycle in patients and controls. Two-way mixed ANOVA (dynPAC ∼ group × period) revealed significant main effects of group and periods, but no significant interaction effects. Post hoc comparisons were performed between neighboring periods (4 tests). *P* values were adjusted by FDR correction across four post hoc tests. Adjusted *P* values below 0.1 are shown in the figure. *D*: slow tapping task. Dynamic PAC resulting from averaging across subjects after alignment with the cortical movement transition point #1. The timing of the other three cortical movement transition points was based on the average duration of the following three periods (T2, T3, and T4) across subjects. *E*: box graph combined with mean lines of movement-related dynamic PAC in the five periods of a single tapping cycle in patients and controls. Two-way mixed ANOVA (dynPAC ∼ group × period) revealed significant interaction effects between group and period, suggesting that dynPAC of patients was modulated differently than that of controls. One-way ANOVA tests for the effect of period were applied to patients and controls separately, both revealed significant effects. Post hoc comparisons were then performed between neighboring periods (4 tests) in patients and controls, respectively. Patients displayed markedly less modulation of PAC than controls. *P* values were then adjusted by FDR correction across 4 post hoc tests. Adjusted *P* values below 0.1 are shown in the figure. The tests in the figure included 19 patients (6 females, mean age: 60.9 ± 10.8 yr) with and 20 healthy controls (8 females, mean age: 62.6 ± 7.9 yr). dynPAC, dynamic PAC; FDI, first dorsal interosseous; FDR, false discovery rate; PAC, phase-amplitude coupling.

We investigated the dynamic PAC (dynPAC) in more detail by looking at the modulation of PAC between five periods (from P1 to P5 in pressing task and from T1 to T5 in slow tapping task) separated by four transition points. The dynamic movement transition points were calculated based on the four kinetic (pressing) or kinematic (tapping) events along the movement cycle (Fig. 5*A*), taking into account mechanical delays, electromechanical delays, and the corticomuscular conduction time to estimate the timing of the transition times between motor states at the level of the cortex (Fig. 5, *B*and *D*). The definition details of the four movement transition points are described in materials and methods, *Dynamic PAC estimation*, as well as in the Supplemental Method “*Definition of 4 transition points of a movement cycle*.”

In the pressing task, PAC was markedly modulated around movement transitions. Figure 5*B* illustrates the averaged dynamics of PAC across the first three periods as resulting from aligning data with the cortical movement transition point #1 (defining the transition between premovement onset and force build-up, *left*), and the last three periods as resulting from aligning data with the cortical movement transition point #4 (defining the transition between force release and press offset, *right*). As shown in Fig. 5*B*, close to the press onset, PAC appeared to decrease from a brief maximum, reaching a minimum before rising again (also evident in Fig. 4). Alignment with the transition at release offset revealed a PAC motif similar to that at press onset (Fig. 5*B*, *right*). Two-way mixed ANOVA on group and period showed significant main effects for the factors group [*F*(1,37) = 5.57, *P* = 0.024] and period [*F*(4,148) = 4.72, *P* = 0.001]. Because there was no group × period interaction effect [*F*(4,148) = 1.08, *P* = 0.369], PAC was not modulated differently between patients and controls. To investigate the effect of period, we tested the difference between each two adjacent periods. As shown in Fig. 5*C*, post hoc testing for the main effect of period indicated that, across subjects, dynPAC decreased significantly during the build-up of the press force compared with the pre-onset period (P1 versus P2). Then, the PAC value rebounded during maintained pressing (P2 vs. P3). In addition, the decrease of dynPAC values from force release to the period after the press offset (P4 vs. P5) showed marginal significance. This finding indicates that dynPAC was modulated across movement transitions before force build-up and after releasing actions.

We also investigated PAC dynamics in slow tapping movement cycles in a manner similar to the pressing task. Since the presence or absence of color feedback had no significant effects on the PAC values in slow tapping, the evaluation of PAC dynamics was based on combining the two conditions. Similar to the pressing task, we found marked modulation of dynPAC in the slow tapping task. As shown in Fig. 5*D*, PAC declined from a brief maximum before index finger extension onset to post extension onset in controls. After reaching a minimum, when the index finger was above the higher amplitude threshold, PAC started to rebound and increased toward the postoffset period. Although the modulation pattern was generally similar in patients, it was markedly flattened compared with controls (also evident in Fig. 4). A two-way mixed ANOVA (dynPAC ∼ group × period) revealed a significant two-way interaction between group and period [*F*(4,148) = 3.59, *P* = 0.008]. This finding indicated that PAC was modulated differently in patients and controls. Therefore, we applied one-way ANOVA on the effect of period for patients and controls, separately. The one-way ANOVA of patients revealed significant effects of period [*F*(4,72) = 3.10, *P* = 0.021, FDR corrected], as did the results for controls [*F*(4,76) = 12.70, *P* < 0.001, FDR corrected]. We then performed post hoc comparisons in terms of differences between adjacent periods, respectively, for patients and controls. As shown in Fig. 5*E*, post hoc testing of neighboring periods of patients revealed that only the PAC decrease from T1 to T2 showed marginal significance. Regarding the PAC changes in controls, dynPAC significantly decreased from T1 to T2, and continuously decreased from T2 to T3, followed by a rebound from T3 to T4, as shown in Fig. 5*E* From the finger-flexion period to the post-flexion offset period (T4 to T5), no significant PAC increase was found in either controls or patients (Fig. 5*E*). Notably, the absolute PAC values did not differ between patients and controls in any of the 5 periods (Wilcoxon rank-sum, all *P* > 0.2). This finding showed less PAC modulation in patients during selected periods of the slow tapping cycle. We subsequently tested the hypothesis that the magnitude of the PAC change around movement onset determines the ability to recruit muscles engaged in the tapping rapidly and thus may contribute to the motor impairment in slow tapping performance in patients. Although the EMG slope was correlated with the PAC change between T1 and T2 in patients, this correlation was lost when the computation of PAC was corrected for the shorter averaged duration of T2 across subjects. EMG slope and PAC change were not significantly correlated in controls (ρ = −0.12, *P* = 0.601).

### Relationship between Movement-Related PAC Dynamics and **β** Power Dynamics

**Figure 6.**
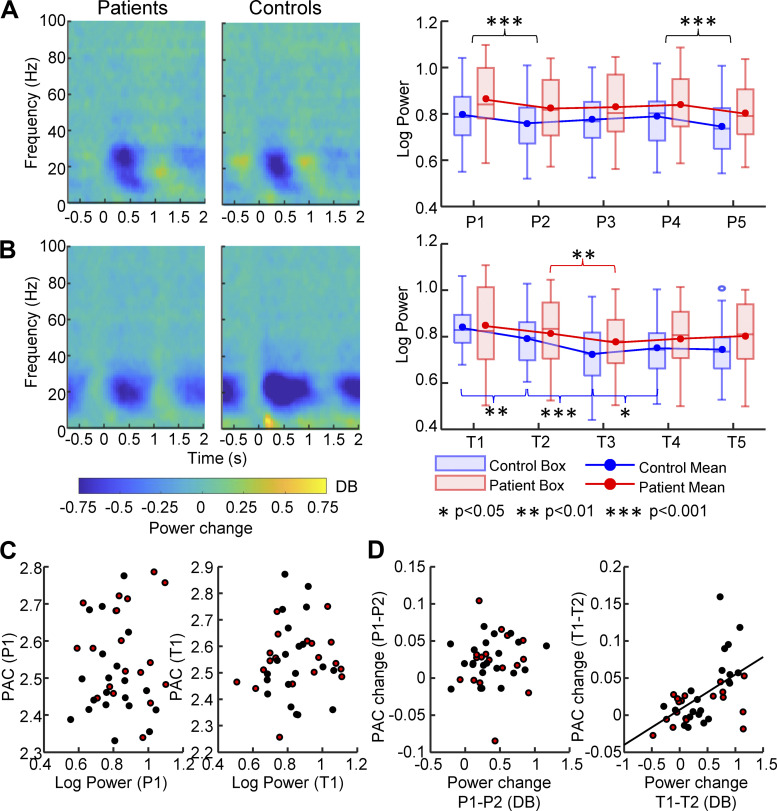
Relationship between movement-related dynPAC and β power. *A*: dynamics of β power in the pressing task. *Left*: time-frequency spectrogram. Note the reduction of β power after the onset of the pressing in both patients and controls. *Right*: box plot combined with mean line graphs show dynamics of β power across the five periods of the pressing movement cycle. Significant modulations (FDR corrected values < 0.05, 0.01, 0.001) are marked by asterisks in the figure. *B*: dynamics of β power in the slow tapping task. *Left*: time-frequency metric plots. *Right*: box plot combined with mean line graphs show the dynamics of β power across the five periods of the slow tapping movement cycle. Significant modulations (FDR corrected values < 0.05, 0.01, 0.001) are marked by asterisks in the figure. *C*: scatter plots of the relationship between the absolute strength of PAC and β power in the pre-onset phase. *D*: scatter plots of the relationship between PAC change and power change from the first to the second period of the movement cycle. *Left*: pressing task. *Right*: slow tapping task. The tests in the figure included 19 patients (6 females, mean age: 60.9 ± 10.8 yr) with and 20 healthy controls (8 females, mean age: 62.6 ± 7. 9 yr). dynPAC, dynamic PAC; FDI, first dorsal interosseous; FDR, false discovery rate; PAC, phase-amplitude coupling.

In the present study, we also found β power to be reduced at movement onset and during movement in both patients and controls (Fig. 6, *A*and *B*, *left*). Because the strength of β power affects the estimation of phases in the calculation of PAC, we investigated to what degree modulation of PAC by movement transitions can be explained by associated changes in β power. We assessed the modulation of β power in the five periods of a movement cycle in the pressing and slow tapping tasks (Fig. 6, *A*and *B*, *right*). The modulation pattern of β power appeared to be similar to the modulation pattern of PAC in both tasks as reported in the result of dynPAC. A two-way mixed ANOVA with group and period was applied to the β power in pressing and slow tapping tasks. The analysis in the pressing task revealed only a main effect of Period [*F*(4,148) = 13.18, *P* < 0.001], while a significant interaction of group and period [*F*(4,148) = 3.98, *P* = 0.004] was revealed in the slow tapping task. This finding raises the question of whether the transient modulation of β power primarily drove PAC modulation during movement. However, we did not find any significant correlation between the absolute PAC and β power values in any of the periods in either the pressing or the slow tapping task (*P* values > 0.3). Two examples of scatter plots between absolute power and PAC in P1 (T1) of pressing (slow tapping) are displayed in Fig. 6*C*. In addition, we performed a correlation analysis between the PAC differences and the β power differences of each set of adjacent periods. At movement onset, the two parameters were not significantly correlated in the pressing task, whereas they were significantly correlated in the slow tapping task (Fig. 6*D*). The correlation results for differences between any two adjacent periods are presented in Table 2, which showed no consistent relationship between β power change and PAC change during the movement. The above findings suggest that the movement-related dynPAC modulation does not generally reflect movement-related power dynamics. However, the fact that derivatives of the two variables were correlated at slow tapping onset could still have pathophysiological significance.

**Table 2.** Relationship between the movement-related change of PAC and change of β power

Task	P1–P2 // T1–T2	P2–P3 // T2–T3	P3–P4 // T3–T4	P4–P5 // T4–T5
Pressing	ρ = 0.13, *P* = 0.429	**ρ = 0.62, *P* = 0.001**	**ρ = 0.42, *P* = 0.017**	ρ = 0.23, *P* = 0.205
Slow tapping	**ρ = 0.50, *P* = 0.003**	**ρ = 0.42, *P* = 0.017**	ρ = −0.09, *P* = 0.568	ρ = 0.18, *P* = 0.362

* *P* values have been FDR corrected for each task, all significant values after correction are marked in bold. FDR, false discovery rate; PAC, phase-amplitude coupling.

A two-way mixed ANOVA with Group and Period was also applied to the γ power in the pressing and slow tapping tasks. However, no interaction effects between group and period were found in either task [pressing: *F*(4,148) = 0.16, *P* = 0.958; slow tapping: *F*(4,148) = 0.14, *P* = 0.967].

## DISCUSSION

The present study examined whether and how cortical β-γ phase-amplitude coupling (PAC) noninvasively recorded during voluntary repetitive movements, is related to motor impairment in PD.

Previous studies have not yielded consistent results on the role of PAC strength during movement in patients with PD with some studies reporting enhanced (13, 15) and at least one other study reporting normal movement-related PAC (14). However, interpretation of these results was hampered by insufficient information about the actual movement performed during the EEG recording. In particular, it remained unclear whether the characteristics of the movements reflected Parkinsonian abnormalities. Our study attempted to address this ambiguity by investigating PAC dynamically and relating its magnitude and modulation to the characteristics of concomitantly performed self-paced repetitive movements. The motor impairment of patients with PD was evident in the reduced EMG slope in the slow tapping task, the lower movement rate in the fast tapping task, and the amplitude decrement during both slow and fast tapping. This is consistent with previous reports (24, 29, 37). However, as assessed by the movement rate and EMG slope, the behavioral performance in a self-paced repetitive pressing and releasing task did not differ between groups. This finding demonstrates that the motor impairment of patients with PD manifests differently across different voluntary repetitive movements. Because the tasks were associated with different probabilities of revealing Parkinsonian motor impairment, we were able to address the question of whether PAC enhancement was a necessary and sufficient condition for Parkinsonian bradykinesia.

In terms of brain activity, we found that movement-related PAC in the pressing task was persistently enhanced in patients compared with controls. This was the case in four cortical areas, all involved in motor control, namely PMC, M1, BA3, and BA1&2. By contrast, we found no differences in the movement-related PAC level between patients and controls in the repetitive tapping tasks, despite the abnormal motor behavior of patients. These results suggest that abnormal enhancement of state-related PAC in movement states is neither necessary nor sufficient for Parkinsonian motor impairment and, therefore, not directly related to the motor symptoms. In addition, our finding that state-related PAC was more strongly suppressed during movements in patients than in controls is consistent with previous suggestions (17) that enhanced resting PAC in patients with PD may return to normal during the movement.

We found that PAC decreased around movement onset in both patients and controls. This reduction of PAC during movement is in line with studies where PAC was derived from oscillatory ECoG signals in M1 (8, 15, 17). It has long been known that voluntary motor activity is also accompanied by event-related β-power desynchronization in the cortex (38–41). In the present study, we found movement-related β power and PAC to be similarly modulated in the slow tapping and pressing tasks. This suggests that the movement-related power and PAC changes were related to a certain degree. Indeed, the suppression of β power could affect the computation of PAC by influencing the estimation of phases. However, the correlation between PAC change and power change among different movement transitions was not always present. Importantly, the magnitude of PAC was not correlated with the absolute β power in previous resting state studies (9, 10, 14) and in the movement states of our study. Furthermore, it has been suggested that cortical PAC is more specifically related to the pathophysiology of PD than is cortical β power. This is evident in DBS and dopamine therapies that improve the motor symptoms of PD by suppressing excessive cortical PAC rather than altering cortical β power (13–15). Therefore, the dynamic modulation of β-γ PAC during repetitive voluntary movements may still encode an essential component of the motor command that is related to the motor impairment of patients with PD. At the same time, it may be distinct from the mechanism underlying event-related desynchronization, even though we cannot exclude some influence of β power on the evaluation of PAC modulation.

Further insight into the role of PAC in motor control was provided by the detailed analysis of its dynamic modulation across a movement cycle (dynPAC). When controls performed the pressing task, PAC exhibited a brief peak followed by a decline during the initial press phase and a subsequent rebound while the index finger maintained constant pressure. Of note, dynPAC showed a similar pattern during the initial releasing phase of the pressing task and around the finger extension onset in the slow tapping task (decrease following a brief peak, followed by a rebound). These findings in healthy controls suggest that PAC decrease is not merely associated with initiating a movement. Rather, there appears to be a characteristic PAC motif (brief peak → decrease → rebound) that signals a change in movement states. This phenomenon resembles the preparatory neuronal activity in the dynamic systems theory of motor control (21). According to this theory, preparatory activity brings the dynamic state of the neuronal population through state-space rotations to an initial value. This process, which is characterized by brief cortical oscillatory activity (20), ensures that muscle activity can be generated efficiently for all types of movements (20, 21). If dynPAC reflects normal preparatory activity, then it is perhaps not surprising that movement-related PAC was found to be similar in patients with PD and patients with essential tremor (17), especially in the absence of kinematic differences between the patient groups.

In patients with PD, we found that dynPAC was abnormal during slow tapping. Although patients attained similar PAC strengths levels before initiating the tapping movements, the subsequent decrease was smaller than that in controls. As was the following rebound. By contrast, during the press and release, dynPAC modulation was similar in patients and controls. These findings appear to be the first to report that abnormal PAC during movement is associated with concurrent abnormal motor performance in PD. If the above proposal that dynPAC is a marker for a preparatory movement state is in fact true, patients with PD may suffer from a defective evolution of a neuronal population dynamic, spanning the preparatory state and overt movement generation. Although PAC would reflect an essential physiological mechanism during the preparation of movements, its persistence into the unfolding movement would interfere with proper execution. Although abnormal dynPAC modulation was associated with slowed muscle recruitment during onset of slow tapping, the magnitude of PAC change did not correlate with the magnitude of the EMG slope. This suggests a complex and non-linear relationship between dynPAC and the build-up of corticospinal neuronal activity. Interestingly, studies probing cortical physiology in the preparatory phase of voluntary movements have provided similar evidence, suggesting that bradykinesia does not result from a single deficient physiological mechanism such as the ability to release ongoing inhibition (42, 43), but reflects a more complex circuit abnormality (44). Notably, in dynamic systems theory of motor control, preparatory activity is sensitive to timing events supporting motor transitions (18). However, it does not reflect specific movement features (e.g., direction, force, velocity), nor does it simply represent the release from inhibition of a motor program (45).

The question arises of why PAC is elevated at rest and why its magnitude is associated with Parkinsonian motor impairment, as reported in previous literature (10, 13). One possibility would be that PAC at rest, or any state-related PAC, is not mechanistically related to the dynPAC abnormality found during slow tapping. Animal experiments may reveal whether state-related PAC and dynPAC could map onto deficient tonic and phasic dopamine activities (46, 47), respectively, which may be tied to deficits in the invigoration of movements (46, 48). If, however, both PAC phenomena are based on a common mechanism, they could reflect processes of movement preparation. In this scenario, on the one hand, the increase of dynPAC would indicate a preparatory state during movement dynamics, with its reduced attenuation at movement transitions indicating a spillover of the preparatory state into the unfolding movement. On the other hand, enhancement of PAC at rest could reflect the abnormal, or abnormally frequent, generation of brief cortical states resembling preparatory population activity without an intention to move. In this way, both the enhanced resting PAC and the reduced dynPAC modulation could be caused by the dysfunction of a single (subcortical) mechanism that controls or regulates the generation of cross-frequency couplings in cortical microcircuits. However, in the absence of direct evidence, the nature of the link between abnormally enhanced PAC at rest and abnormal modulation of dynPAC must remain a topic for future investigations.

### Limitations

Although self-paced tapping movements may be considered relatively elementary, and although they did reflect the motor impairment of PD in our study, different mechanisms may underlie performance impairment in other types of movement. Therefore, future studies need to explore abnormal cross-frequency coupling more comprehensively during other variable motor behaviors.

The resolution of PAC computation across the movement cycle is constrained by the minimum number of oscillatory cycles, which in turn, depends on the involved oscillation frequencies. Therefore, dynamic modulation of PAC cannot be resolved by the current analytic techniques during fast repetitive movements. In addition, separation of the movement cycles into discrete periods may not apply to everyday behavior, which in many instances is more appropriately conceived of as continuous action with no discrete transitions. It remains to be studied whether concepts derived from analyzing movement periods translate into the control of continuous movements.

Finally, patients with marked resting tremor were excluded and all recordings were done in patients with early to moderate disease stages. Therefore, it remains unclear how generalizable our findings are to tremulous or more severe PD phenotypes.

### Conclusions

In conclusion, the present study provides evidence that the strength of PAC during movement is not directly related to the motor impairment of patients with PD. Instead, the association of abnormal PAC dynamics with bradykinesia is compatible with the hypothesis that deficient dynamic regulation of PAC is causally involved in the pathophysiology of Parkinsonian motor impairment.

## SUPPLEMENTAL DATA

10.5281/zenodo.6535236 The supplementary material is available through the link https://doi.org/10.5281/zenodo.6535236. This file includes Supplementary Method, Supplementary Figure, and Supplementary Table.

## GRANTS

This work was supported by the CortExplorer program (P1140048) of Hertie Foundation (Gemeinnützige HERTIE-Stiftung) (to J.C.). R.G. is the recipient of a scholarship of the International Max Planck Research Schools (IMPRS) NeuroCom program.

## DISCLOSURES

No conflicts of interest, financial or otherwise, are declared by the authors.

## AUTHOR CONTRIBUTIONS

R.G., C.F., J.-J.R., T.R.K., and J.C. conceived and designed research; R.G., C.M., and M.W. performed experiments; R.G., T.R.K., and J.C. analyzed data; R.G., T.R.K., and J.C. interpreted results of experiments; R.G. and J.C. prepared figures; R.G. drafted manuscript; R.G., C.M., M.W., C.F., J.-J.R., T.R.K., and J.C. edited and revised manuscript; R.G., C.M., M.W., C.F., J.-J.R., T.R.K., and J.C. approved final version of manuscript.
